# Risk factors for clinical failure of peroral endoscopic myotomy in achalasia

**DOI:** 10.3389/fmed.2022.1099533

**Published:** 2022-12-08

**Authors:** Lucille Quénéhervé, Blandine Vauquelin, Arthur Berger, Emmanuel Coron, Raphael Olivier

**Affiliations:** ^1^Gastroenterology Department, University Hospital of Brest, Brest, France; ^2^Gastroenterology Department, Centre Medico-Chirurgical Magellan, INSERM CIC 1401, CHU de Bordeaux, Hôpital Haut-Lévêque, Université de Bordeaux, Bordeaux, France; ^3^Service de Gastro-Entérologie et Hépatologie, Hôpitaux Universitaires de Genève, Geneva, Switzerland; ^4^Gastroenterology Department, University Hospital of Poitiers, Poitiers, France

**Keywords:** achalasia, per oral endoscopic myotomy (POEM), clinical success, risk factors, predictive score

## Abstract

The recent development of per oral endoscopic myotomy (POEM) has been a game changer in the management of patients with achalasia. However, approximately 1 in 10 patients will not experience clinical success. The aim of this mini-review is to describe the current state of knowledge about the risk factors associated with POEM failure for the treatment of achalasia. Suspected risk factors are detailed into pre-, intra-, and post-procedural factors and put into perspective. Pre-procedural factors have been described, such as pre-treatment Eckardt score, previous treatments for achalasia, sigmoid type esophagus, significant esophageal dilatation, non-type II achalasia, young age and long duration of symptoms. An intra-procedural factor, mucosal injury during POEM, has also been associated with POEM failure. The occurrence of post-POEM GERD was identified as a controversial post-procedural factor associated with failure. The presumed mechanisms of POEM failure are incomplete myotomy or ineffective LES disruption, as confirmed by high-resolution manometry. However, when manometry confirms a significant decrease in LES pressure, it is likely that either impaired peristalsis or a morphologic abnormality such as extreme esophageal dilatation or severe tortuosity, which are not treated by POEM, should be suspected. Notably, a recently described adverse effect of POEM is the formation of a pseudo-diverticulum at the site of the myotomy (blown out myotomy). We finally stress the importance of performing a complete workup in case of POEM failure as different mechanisms of POEM failure should lead to different management.

## Introduction

The recent development of Per oral endoscopic myotomy (POEM) has been a game changer in the management of patients with achalasia. POEM involves the creation of a submucosal tunnel in the esophagus wall using a standard gastroscope, allowing myotomy of the circular muscle layer to reduce pressure at the lower esophageal sphincter (LES) and then closing of the tunnel to protect the myotomy site from infection (1). It is primarily used to treat achalasia, a functional condition of the esophagus, characterized by the combination of failed esophageal peristalsis and lack of relaxation of the LES, as reflected by a high integrated relaxation pressure on high-resolution manometry (2). Over the past 12 years and despite its innovating concept, POEM has grown dramatically due to its excellent clinical results and tolerance (3, 4). However, short-term clinical success is not achieved in approximately 1 in 10 patients (5, 6).

The mini review aims to describe the current state of knowledge about the risk factors associated with POEM failure in the treatment of achalasia.

## Definition of clinical failure

The Eckardt score is the most widely used symptom score in achalasia; it was developed to assess clinical response to treatment, at the time pneumatic dilation (7). It consists of 4 items, namely weight loss, dysphagia, retrosternal pain, and regurgitation, each scored from 0 to 3. It has been suggested that the Eckardt score could be refined, as retrosternal pain, which is inconstant in patients with achalasia, and weight loss, which is multifactorial, appear to be less reliable than other factors (8). In most studies evaluating the efficacy of achalasia treatment, clinical failure is defined by an Eckardt score > 3 (6, 9–11) but other outcomes such as other scores or the need for further treatment have been used (12).

## Risk factors

Suspected risk factors can be classified into pre-procedural, intra-procedural, and post-procedural factors (Table 1).

**TABLE 1:** Risk factors for clinical failure of peroral endoscopic myotomy in achalasia.

	Putative factors	Level of risk
**Pre-procedural factors**		
	Pre-treatment Eckardt score	+ ++
	Long disease duration	+ +
	Prior achalasia treatment	+ ++
	Dilated esophagus and sigmoid-shaped esophagus	+ +
	Non-type II achalasia	+ ++
	Young age	±
	Sex	−
	Cardiac muscle thickness	+
**Intra-procedural factors**		
	Location of the myotomy	−
	Length of the myotomy	−
	Experience of the operator	+ +
	Mucosal injury	+
**Post-procedural factors**		
	Gastro-esophageal reflux	±

(+ + +) probable; (+ +) likely; (+) possible; (±) conflicting data; (−) unlikely.

### Pre-procedural factors

Several pre-procedural factors have been described that may be used to better inform patients and select the best treatment options.

The pre-treatment Eckardt score was identified as a predicting factor for failure in a prospective Chinese study of 115 patients (odd-ratio (OR) 2.24) (13). A score ≥ 9 was associated with high sensitivity and specificity in predicting POEM failure. This was confirmed in 2 Japanese studies that identified a positive association of pretreatment Eckardt score with failure (OR 1.17 to 1.45 for a one-point increment in preprocedural Eckardt score) (14, 15).

Conversely, a Chinese retrospective study of 564 patients (16) found no impact of pre-treatment Eckardt score but identified long disease duration (≥ 10 years) as a risk factor with an hazard ratio (HR) of 2.45. Similarly, a Norwegian series showed that symptom duration of 5 years and more had a negative impact on POEM outcome (OR 6.67) (17).

A previous treatment for achalasia was associated with POEM failure, with the risk increasing from 1.12 to 3.75 in Asian studies (15, 16, 18). Another study found that the risk of POEM failure was greater in case of prior Heller’s myotomy (OR 4.55; reference: no previous treatment) than when patients had a history of pneumatic dilation. One explanation could be that patients with prior treatment often have a longer duration of disease and are more likely to have sigmoid esophagus and submucosal fibrosis (19). Submucosal fibrosis at the gastroesophageal junction caused by previous endoscopic treatment, i.e., botulinum toxin injection, pneumatic dilation or a first POEM, or surgical myotomy, could increase the difficulty of submucosal dissection. In addition, patients with prior treatment may have esophageal motility disorders that are inherently refractory to treatment. However, data are conflicting, with several studies demonstrating the safety and efficacy of POEM in non-naïve patients (20, 21).

Dilated esophagus and sigmoid shaped esophagus were found to be risk factors for POEM failure. Urakami et al. found that sigmoid-type esophagus, characterized by subsequent tortuous angulation <135° of esophageal lumen (22), and esophageal dilation grade ≥ II, i.e., with a diameter >3.5 cm, were associated with POEM failure (OR of 3.68 and 3.75 respectively) (15). Similarly, an achalasia stage of II or higher, i.e., esophageal diameter >3 cm or sigmoid esophagus (23), was associated with failure assessed by timed barium esophagogram in the series by Evensen et al. (OR 10.6) (17). As esophagogram is less sensitive than high-resolution manometry (24), it is not always performed during achalasia workup, therefore esophageal diameter and angulation are not routinely measured, which might explain why these parameters do not appear in all achalasia series. The role of esophageal morphology in POEM failure is probably related to the persistence of delayed esophageal transit in dilated esophagus, even after LES myotomy (25). However, esophageal morphology was not an independent factor in a large Japanese series, probably because it is closely related to disease duration, history of previous treatment and the type of achalasia (14).

The type of achalasia also seems to play a role, with type II achalasia having the most favorable profile compared with types I and III. A meta-analysis (12) confirmed that POEM was more likely to achieve clinical success than surgical treatment in type I and type III (OR 2.97 and 3.50, respectively). However, this analysis also reported that clinical success after POEM was achieved in 95% of patients with type I achalasia, 97% of those with type II and 93% of those with type III, highlighting both the excellent results of POEM and the better prognosis of type II achalasia. Another meta-analysis (26) showed inconclusive results regarding the impact of achalasia subtypes. Type III achalasia is less common than type I and II; in a Japanese multicenter study (14) types I, II, and III accounted for 55.4%, 38.9% and 5.7% of achalasia cases, respectively. The favorable profile of type II achalasia may be due to pressurization which improves esophageal emptying after treatment (27).

Young age is often considered a risk factor for POEM failure, by analogy with pneumatic dilation. A series from Western countries showed that clinical success was increased by a factor of 1.6 per 10 years (28). However, a meta-analysis exploring the risk factors for clinical failure of different achalasia treatments, while confirming that age was a risk factor for failure of pneumatic dilation, found no relationship between age and POEM failure (26). In the same study, sex was not associated with POEM failure.

Cardiac muscle thickness was associated with POEM outcome in a Chinese retrospective study in which an endoscopic ultrasound examination was performed before POEM in patients with achalasia (29). Thin muscle (< 3mm) was associated with more frequent POEM failure than thick muscle.

Panometry profile using functional luminal imaging probe (FLIP) has not been associated with POEM outcomes but the level of evidence remains low (30).

### Intra-procedural factors

As they are directly dependent on the procedure, these are the factors on which the operator can have the most influence.

Technical factors such as the orientation of the myotomy (anterior or posterior) (31, 32) as well as the length of the myotomy (33) do not seem to have an impact on the clinical outcome of POEM in prospective series. The experience of the operator could also be associated with POEM outcome but there are conflicting data on how many POEM cases must be performed to be competent (34).

Mucosal injury during POEM has been associated with POEM failure (18). In this Chinese study mucosal injury were divided into two categories: stage I, i.e., small superficial mucosal injuries, that could be easily repaired, and type II, i.e., large full-thickness perforations with an irregular border, which were difficult to repair. Compared with no mucosal injury, stage II mucosal injuries were associated with a higher risk of POEM failure (HR 6.35; p < 0.001), whereas there was only a trend for stage I injuries. The authors hypothesized that wound scarring may induce re-constriction of the LES.

### Post-procedural factors

Post procedural factors cannot be used to plan the patient management but would be early predictors of POEM failure. To our knowledge, only one, post-POEM gastro-esophageal reflux (GERD) has been discussed but its role remains controversial, as an adverse event inherently linked to the decrease in LES that is induced and sought by POEM. Therefore, the occurrence of post-POEM GERD should not be considered a failure *per se* but different teams have investigated whether it could be related to the effectiveness of POEM. Clinical GERD, defined at either symptomatic reflux assessed by a questionnaire or esophagitis, was identified by Liu and colleagues (18) as a post-procedural factor associated with failure (OR 3.01). However, endoscopic evidence of GERD 3 to 6 months after treatment was associated with POEM clinical success in another study (OR 6.76) (28).

## Scoring systems

Several recent initiatives to develop a scoring system for the risk of POEM failure have been published (Supplementary material).

First, in a Chinese single-center retrospective study (18) a point-scoring system was developed by assigning 2 points to prior treatment, 2 points to type I mucosal injury, 6 points to type II mucosal injury, and 3 points to clinical GERD. In the validation cohort, patients with 4 points or more had a 4-fold higher risk of POEM failure than patients with fewer than 4 points. However, this score, which encompasses pre-, intra- and post- procedural variables cannot be used pre-operatively and therefore cannot guide early patient management.

Second, in a Japanese single-center retrospective study (15) designed to predict clinical failure of POEM for esophageal motility disorders, risk points were assigned for pretreatment Eckardt score (1 point for a one-point increment in the preprocedural Eckardt score), previous treatments (4 points), sigmoid-type esophagus (4 points), and esophageal dilation grade ≥ II (4 points). In the low-risk group (<10 points), intermediate-risk group (10 to 15 points), and high-risk group (>15 points) the percentage of poor responders was 6.6, 16.3 and 66.7%, respectively. One limitation is that the type of achalasia was not analyzed although it is likely to be an important predictor.

Third, a large Japanese multicenter case-control study (14) developed a score to predict clinical failure of POEM for esophageal motility disorders, with non-achalasia motility disorders grouped with type III achalasia in “non-type I/II achalasia.” Three preoperative factors were identified in a multivariate analysis and points were assigned accordingly: preprocedural Eckardt score (1 point for a one-point increment), manometric diagnosis (–4 points for type II achalasia), and previous treatments (1 point for pneumatic dilation or 12 points for surgical/endoscopic myotomy). Risk was categorized as low when the score was <9 points, with an estimated risk <5%, and high when the score was ≥9 points with an estimated risk ≥5%. The discrimination capacity of this promising score was not considered sufficiently robust by the authors, who hypothesized that other predictors remain to be identified.

Most studies on predictors of POEM failure have been conducted in Asian patients and data are needed regarding risk factors in Western countries (35).

## Mechanisms of per oral endoscopic myotomy failure

Various hypotheses have been discussed regarding the mechanisms of POEM failure. The main presumed mechanism of POEM failure is incomplete myotomy or ineffective LES disruption, as confirmed by high-resolution manometry. However, when manometry confirms a significant decrease in LES pressure, it is likely that either impaired peristalsis or a morphologic abnormality such as extreme esophageal dilatation or severe tortuosity, which are not treated by POEM, should be suspected (36). Notably, a recently described adverse effect of POEM is the formation of a pseudo-diverticulum at the site of myotomy (blown out myotomy-BOM) (Supplementary material). In a US study, esophagograms of patients who underwent surgical myotomy or POEM for achalasia were examined to assess the presence of a BOM, defined as a distal wide-mouthed (>2 cm) diverticulum in the area of the prior myotomy with more than a 50% increase in esophageal diameter, potentially favorized by a weakness in the esophageal muscle due to the myotomy. Myotomy failure was more common in patients with BOM, which was associated with type III achalasia, high post treatment integrated relaxation pressure and surgical myotomy (37). The authors hypothesized that BOM could be prevented by a shorter myotomy in type I and II achalasia and that fundoplication by increasing pressure above the gastroesophageal junction might increase the risk of BOM (38).

Finally, other causes of esophageal symptoms must be investigated when Eckardt score fails to decrease after POEM.

A better understanding of the pathophysiology of achalasia would allow to adapt the treatment, which can be considered as palliative at present. Current theories on achalasia were apprehended through the study of esophagectomy specimens but several teams have attempted to take advantage of the myotomy tunnel to sample muscle to study the mechanisms leading to achalasia (39, 40).

## Management of per oral endoscopic myotomy failure

The different mechanisms of POEM failure should lead to different management.

**FIGURE 1:**
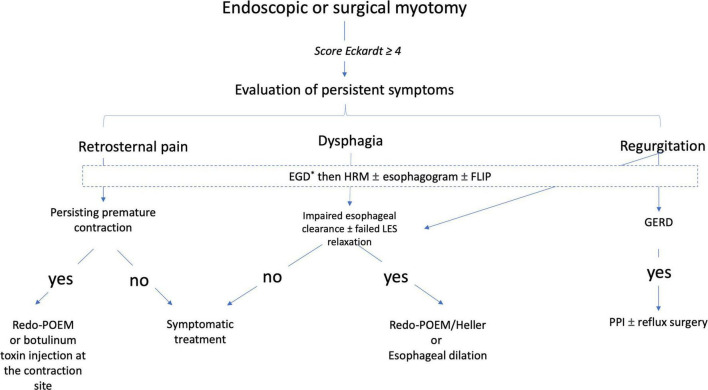
A proposed algorithm of management after failure of an endoscopic or surgical myotomy for the treatment of achalasia (Expert opinion). *Search by esogastroduodenoscopy for esophagitis and peptic stenosis. EGD, esogastroduodenoscopy; FLIP, functional luminal imaging probe; GERD, gastroesophageal reflux; HRM, high-resolution manometry; PPI, proton pump inhibitors.

After the careful interview of the patient and treatment of a potential GERD, a complete workup should be performed, including upper GI endoscopy, manometry, pH study and esophagogram. The role of FLIP has also been advocated in this indication (41). The type of persistent symptoms should be investigated, with retrosternal pain being the most difficult to treat. If the integrated relaxation pressure is still elevated, incomplete myotomy is likely and retreatment such as repeat POEM or pneumatic dilation may be advocated (42). When upper GI endoscopy demonstrates esophageal stricture, especially in patients experiencing symptoms of GERD, hydrostatic dilation can be performed. Options for patients with low integrated relaxation pressure and endoscopy and esophagogram showing esophageal distension with food stasis are still limited. The authors suggest an algorithm for the management of persistent symptoms after endoscopic or surgical myotomy based on their experience (Figure 1).

Patients should be informed by their physician that all achalasia-related symptoms may not disappear after POEM since the esophagus body remains abnormal.

## Conclusion

Per oral endoscopic myotomy is an excellent treatment of achalasia, which has rapidly taken over the world. Long-term studies show a slight decrease in clinical success over time (43). Large-scale studies are needed to confirm identified risk factors and find new ones. Identification of the mechanisms and risk factors for POEM failure will allow physicians to tailor patient management according to their identified risk and improve the operator practices.

## Author contributions

LQ drafted the manuscript. BV, AB, EC, and RO performed a critical review of the manuscript. AB provided the figure. All authors contributed to the article and approved the submitted version.

## References

[1] Inoue H Minami H Kobayashi Y Sato Y Kaga M Suzuki M Peroral endoscopic myotomy (POEM) for esophageal achalasia. *Endoscopy.* (2010) 42:265–71. 10.1055/s-0029-1244080 20354937

[2] Yadlapati R Kahrilas PJ Fox MR Bredenoord AJ Prakash Gyawali C Roman S Esophageal motility disorders on high-resolution manometry: Chicago classification version 4.0©. *Neurogastroenterol Motil.* (2021) 33:e14058. 10.1111/nmo.14058 PMC8034247 33373111

[3] Akintoye E Kumar N Obaitan I Alayo QA Thompson CC. Peroral endoscopic myotomy: a meta-analysis. *Endoscopy.* (2016) 48:1059–68. 27617421 10.1055/s-0042-114426

[4] Haito-Chavez Y Inoue H Beard KW Draganov PV Ujiki M Rahden BHA Comprehensive analysis of adverse events associated with per oral endoscopic myotomy in 1826 patients: an international multicenter study. *Am J Gastroenterol.* (2017) 112:1267–76. 10.1038/ajg.2017.139 28534521

[5] Inoue H Sato H Ikeda H Onimaru M Sato C Minami H Per-oral endoscopic myotomy: a series of 500 patients. *J Am Coll Surg.* (2015) 221:256–64. 10.1016/j.jamcollsurg.2015.03.057 26206634

[6] Modayil RJ Zhang X Rothberg B Kollarus M Galibov I Peller H Peroral endoscopic myotomy: 10-year outcomes from a large, single-center U.S. series with high follow-up completion and comprehensive analysis of long-term efficacy, safety, objective GERD, and endoscopic functional luminal assessment. *Gastrointest Endosc.* (2021) 94:930–42. 10.1016/j.gie.2021.05.014 33989646

[7] Eckardt VF Aignherr C Bernhard G. Predictors of outcome in patients with achalasia treated by pneumatic dilation. *Gastroenterology.* (1992) 103:1732–8. 1451966 10.1016/0016-5085(92)91428-7

[8] Taft TH Carlson DA Triggs J Craft J Starkey K Yadlapati R Evaluating the reliability and construct validity of the Eckardt symptom score as a measure of achalasia severity. *Neurogastroenterol Motil.* (2018) 30:e13287. 10.1111/nmo.13287 29315993 PMC5992017

[9] Boeckxstaens GE Annese V Varannes SB Chaussade S Costantini M Cuttitta A Pneumatic dilation versus laparoscopic Heller’s myotomy for idiopathic achalasia. *N Engl J Med.* (2011) 364:1807–16. 21561346 10.1056/NEJMoa1010502

[10] Werner YB Hakanson B Martinek J Repici A von Rahden BHA Bredenoord AJ Endoscopic or surgical myotomy in patients with idiopathic achalasia. *N Engl J Med.* (2019) 381:2219–29. 31800987 10.1056/NEJMoa1905380

[11] Ponds FA Fockens P Lei A Neuhaus H Beyna T Kandler J Effect of peroral endoscopic myotomy vs pneumatic dilation on symptom severity and treatment outcomes among treatment-naive patients with achalasia: a randomized clinical trial. *JAMA.* (2019) 322:134–44. 31287522 10.1001/jama.2019.8859 PMC6618792

[12] Andolfi C Fisichella PM. Meta-analysis of clinical outcome after treatment for achalasia based on manometric subtypes. *Br J Surg.* (2019) 106:332–41. 10.1002/bjs.11049 30690706

[13] Ren Y Tang X Chen Y Chen F Zou Y Deng Z Pre-treatment Eckardt score is a simple factor for predicting one-year peroral endoscopic myotomy failure in patients with achalasia. *Surg Endosc.* (2017) 31:3234–41. 10.1007/s00464-016-5352-5 27864723

[14] Abe H Tanaka S Sato H Shimamura Y Okada H Shiota J Risk scoring system for the preprocedural prediction of the clinical failure of peroral endoscopic myotomy: a multicenter case-control study. *Endoscopy.* (2022) [Online ahead of print]. 10.1055/a-1876-7554 35705149

[15] Urakami S Abe H Tanaka S Kawara F Toyonaga T Ariyoshi R Development of a preoperative risk-scoring system for predicting poor responders to peroral endoscopic myotomy. *Gastrointest Endosc.* (2021) 93:398–405. 10.1016/j.gie.2020.06.028 32565185

[16] Li QL Wu QN Zhang XC Xu MD Zhang W Chen SY Outcomes of per-oral endoscopic myotomy for treatment of esophageal achalasia with a median follow-up of 49 months. *Gastrointest Endosc.* (2018) 87:1405–12.e3. 29108981 10.1016/j.gie.2017.10.031

[17] Evensen H Småstuen MC Schulz A Kristensen V Larssen L Skattum J One year comprehensive prospective follow-up of achalasia patients after peroral endoscopic myotomy. *Ann Med.* (2021) 53:2227–35. 10.1080/07853890.2021.2005253 34806501 PMC8805855

[18] Liu XY Cheng J Chen WF Liu ZQ Wang Y Xu MD A risk-scoring system to predict clinical failure for patients with achalasia after peroral endoscopic myotomy. *Gastrointest Endosc.* (2020) 91:33–40.e1. 10.1016/j.gie.2019.07.036 31421076

[19] Liu ZQ Li QL Chen WF Zhang XC Wu QN Cai MY The effect of prior treatment on clinical outcomes in patients with achalasia undergoing peroral endoscopic myotomy. *Endoscopy.* (2019) 51:307–16. 10.1055/a-0658-5783 30261536

[20] Nabi Z Ramchandani M Chavan R Tandan M Kalapala R Darisetty S Peroral endoscopic myotomy in treatment-naïve achalasia patients versus prior treatment failure cases. *Endoscopy.* (2018) 50:358–70. 10.1055/s-0043-121632 29169196

[21] Orenstein SB Raigani S Wu YV Pauli EM Phillips MS Ponsky JL Peroral endoscopic myotomy (POEM) leads to similar results in patients with and without prior endoscopic or surgical therapy. *Surg Endosc.* (2015) 29:1064–70. 10.1007/s00464-014-3782-5 25249143

[22] Japan Esophageal Society. Descriptive rules for achalasia of the esophagus, June 2012: 4th edition. *Esophagus.* (2017) 14:275–89. 10.1007/s10388-017-0589-1 28983228 PMC5603650

[23] Patel KS Calixte R Modayil RJ Friedel D Brathwaite CE Stavropoulos SN. The light at the end of the tunnel: a single-operator learning curve analysis for per oral endoscopic myotomy. *Gastrointest Endosc.* (2015) 81:1181–7. 10.1016/j.gie.2014.10.002 25597422

[24] Oude Nijhuis R Zaninotto G Roman S Boeckxstaens GE Fockens P Langendam MW European guidelines on achalasia: United European gastroenterology and european society of neurogastroenterology and motility recommendations. *United European Gastroenterol J.* (2020) 8:13–33. 10.1177/2050640620903213 32213062 PMC7005998

[25] Rhee K Jeon H Kim JH Yoon YH Park H Lee SI. An evidence of esophageal decompensation in patients with achalasia in the view of its subtype: a retrospective study. *J Neurogastroenterol Motil.* (2013) 19:319–23. 10.5056/jnm.2013.19.3.319 23875098 PMC3714409

[26] Oude Nijhuis RAB Prins LI Mostafavi N van Etten-Jamaludin FS Smout AJPM Bredenoord AJ. Factors associated with achalasia treatment outcomes: systematic review and meta-analysis. *Clin Gastroenterol Hepatol.* (2020) 18:1442–53. 10.1016/j.cgh.2019.10.008 31622735

[27] Hong SJ Bhargava V Jiang Y Denboer D Mittal RK. A unique esophageal motor pattern that involves longitudinal muscles is responsible for emptying in achalasia esophagus. *Gastroenterology.* (2010) 139:102–11. 10.1053/j.gastro.2010.03.058 20381493 PMC2950263

[28] Werner YB Costamagna G Swanström LL von Renteln D Familiari P Sharata AM Clinical response to peroral endoscopic myotomy in patients with idiopathic achalasia at a minimum follow-up of 2 years. *Gut.* (2016) 65:899–906. 10.1136/gutjnl-2014-308649 25934759

[29] He QL Chen XB Lu DH Luo W Tao L Ning HJ The relationship between cardiac muscularis propria and clinical outcomes of peroral endoscopic myotomy in achalasia. *Clin Res Hepatol Gastroenterol.* (2021) 45:101529. 10.1016/j.clinre.2020.08.007 33268035

[30] Hsing LC Choi K Jung KW Joo S Kim N Kim GH The predictive value of intraoperative esophageal functional luminal imaging probe panometry in patients with achalasia undergoing peroral endoscopic myotomy: a single-center experience. *J Neurogastroenterol Motil.* (2022) 28:474–82. 10.5056/jnm21186 35799241 PMC9274461

[31] Khashab MA Sanaei O Rivory J Eleftheriadis N Chiu PWY Shiwaku H Peroral endoscopic myotomy: anterior versus posterior approach: a randomized single-blinded clinical trial. *Gastrointest Endosc.* (2020) 91:288–97.e7. 10.1016/j.gie.2019.07.034 31408652

[32] Ichkhanian Y Abimansour JP Pioche M Vosoughi K Eleftheriadis N Chiu PWY Outcomes of anterior versus posterior peroral endoscopic myotomy 2 years post-procedure: prospective follow-up results from a randomized clinical trial. *Endoscopy.* (2021) 53:462–8. 10.1055/a-1204-4242 32572862

[33] Nabi Z Talukdar R Mandavdhare H Reddy DN. Short versus long esophageal myotomy during peroral endoscopic myotomy: a systematic review and meta-analysis of comparative trials. *Saudi J Gastroenterol.* (2022) 28:261–7. 10.4103/sjg.sjg_438_21 34806659 PMC9408737

[34] Puli SR Wagh MS Forcione D Gopakumar H. Learning curve for esophageal peroral endoscopic myotomy: a systematic review and meta-analysis. *Endoscopy.* (2022) [Online ahead of print]. 10.1055/a-1935-1093 36049775

[35] Vauquelin B Berger A Pioche M Barret M. Risk factors for early failure of peroral endoscopic myotomy (poem) in achalasia: a retrospective multicenter study. *Endoscopy.* (2022) 54:S8.

[36] Jain AS Carlson DA Triggs J Tye M Kou W Campagna R Esophagogastric junction distensibility on functional lumen imaging probe topography predicts treatment response in achalasia-anatomy matters! *Am J Gastroenterol.* (2019) 114:1455–63. 10.14309/ajg.0000000000000137 30741739 PMC6682473

[37] Triggs JR Krause AJ Carlson DA Donnan EN Campagna RAJ Jain AS Blown-out myotomy: an adverse event of laparoscopic Heller myotomy and peroral endoscopic myotomy for achalasia. *Gastrointest Endosc.* (2021) 93:861–8.e1. 10.1016/j.gie.2020.07.041 32721488 PMC7855725

[38] Halder S Acharya S Kou W Campagna RAJ Triggs JR Carlson DA Myotomy technique and esophageal contractility impact blown-out myotomy formation in achalasia: an in silico investigation. *Am J Physiol Gastrointest Liver Physiol.* (2022) 322:G500–12. 10.1152/ajpgi.00281.2021 35170365 PMC8993593

[39] Liu X Kuo E Wang K Perbtani YB Yang D Draganov P. Histologic findings in mucosa and muscularis propria biopsied during peroral endoscopic myotomy in patients with achalasia. *Gastroenterology Res.* (2021) 14:281–9. 34804272 10.14740/gr1454 PMC8577593

[40] Chen H Calderon LF Shah R Zheng W Xia L Wang W Simultaneous examination of eosinophil infiltration in esophageal mucosa and muscle in patients with achalasia: direct biopsy of the esophageal muscle at per-oral endoscopic myotomy. *Dig Dis Sci.* (2022) 67:170–6. 10.1007/s10620-021-06827-4 33502676 PMC7838844

[41] Ichkhanian Y Brewer Gutierrez O Roman S Yoo IK Canakis A Pawa R Role of functional luminal imaging probe in the management of postmyotomy clinical failure. *Gastrointest Endosc.* (2022) 96:9–17.e3. 10.1016/j.gie.2022.02.002 35149045

[42] Ichkhanian Y Assis D Familiari P Ujiki M Su B Khan SR Management of patients after failed peroral endoscopic myotomy: a multicenter study. *Endoscopy.* (2021) 53:1003–10. 33197943 10.1055/a-1312-0496

[43] Vespa E Pellegatta G Chandrasekar VT Spadaccini M Patel H Maselli R Long-term outcomes of peroral endoscopic myotomy for achalasia: a systematic review and meta-analysis. *Endoscopy.* (2022). 10.1055/a-1894-0147 [Epub ahead of print]. 35798336

