# Supplementary material for: Risk factors for clinical failure of peroral endoscopic myotomy in achalasia
Source: Front Med (Lausanne). 2022 Dec 8;9:1099533. doi: 10.3389/fmed.2022.1099533 (PMC9773253; doi:10.3389/fmed.2022.1099533)

Supplementary Table 1: Predictive scores for POEM failure

Article	Design	Scoring system	Risk of POEM failure
Liu et al., 2020 (18)	Single-center, retrospective study of 2367 consecutive patients diagnosed with achalasia and treated with POEM	- previous treatment (+2)<br>- type I mucosal injury (+2)<br>- type II mucosal injury (+6)<br>- clinical GERD (+3)	- Low-risk group (<4 points): 5%<br>- High-risk group (≥4 points): 20%<br>(validation cohort)
Urakami et al., 2021 (15)	Single-center, retrospective study of 244 consecutive patients diagnosed with esophageal motility disorders and treated with POEM	- pretreatment Eckardt score (+1 for a one-point increment in the preprocedural score)<br>- previous treatments (+4)<br>- sigmoid-type esophagus (+4)<br>- esophageal dilation grade ≥II (+4)	- Low-risk group (<10 points): 6.6%<br>- Intermediate-risk group (10 to 15 points): 16.3%<br>- High-risk group (>15 points): 66.7%
Abe et al. 2022(14)	Multicenter retrospective study on 2740 consecutive patients diagnosed with esophageal motility disorders and treated with POEM	- preprocedural Eckardt score (+1 for a one-point increment)<br>- manometric diagnosis (−4 for type II achalasia)<br>- previous treatments (+1 for pneumatic dilation, +12 points for surgical/endoscopic myotomy).	- Low-risk group (<9 points): <5%<br>- High-risk group (≥9 points): ≥5%.

Figure 1: Blown-out myotomy. Illustration of the mechanism of BOM (A); High resolution manometry showing a typical post-myotomy appearance with disappearance of lower esophageal sphincter pressure (B); Right anterior oblique incidence esophagogram showing esophageal deformation observed in BOM (C); Endoscopic view of a pseudo-diverticulum above the squamocolumnar junction (arrow) observed in case of BOM (D).

A

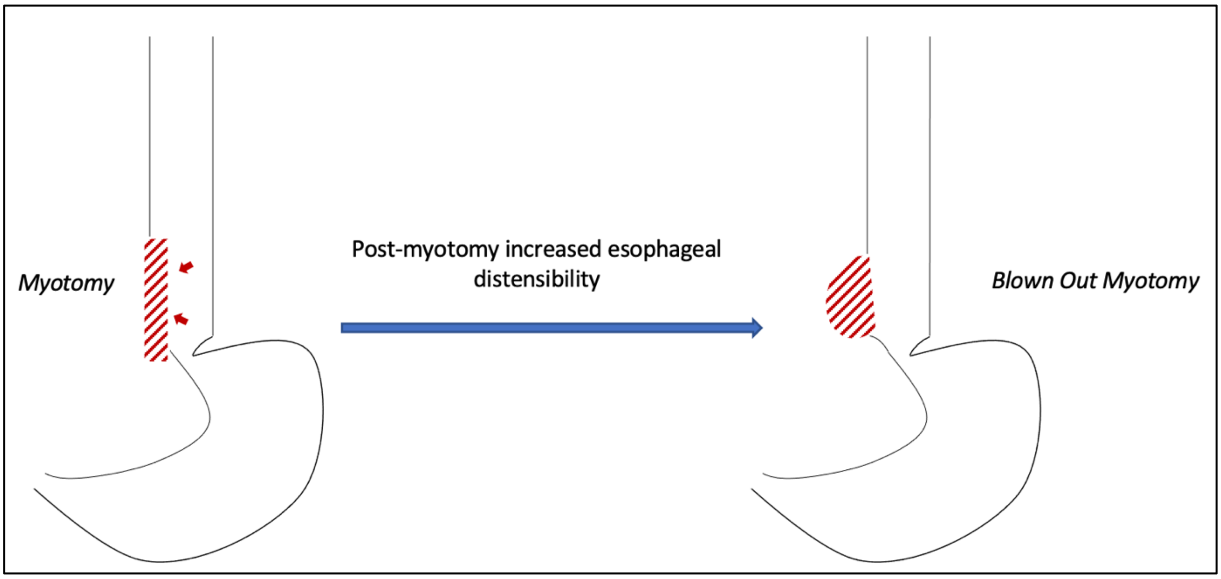

B

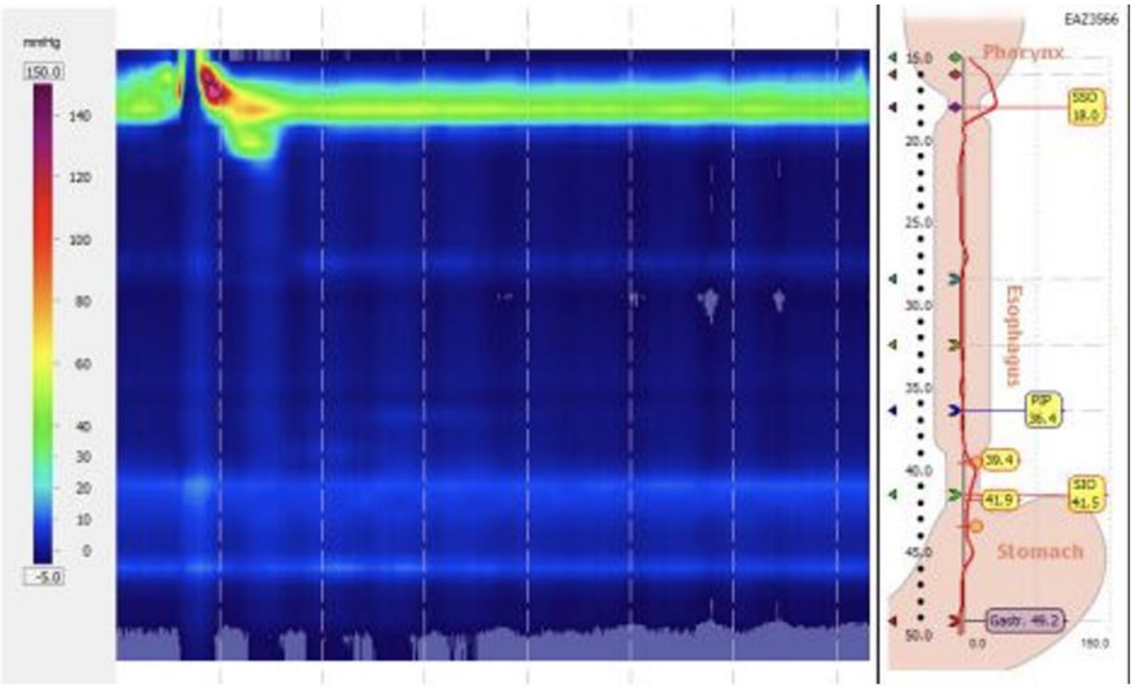

C

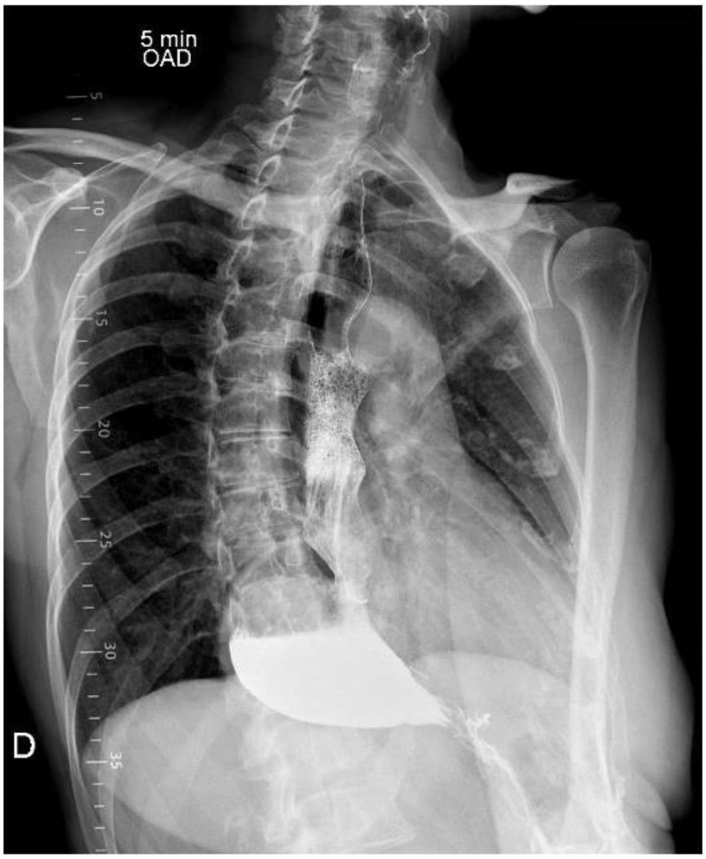

D

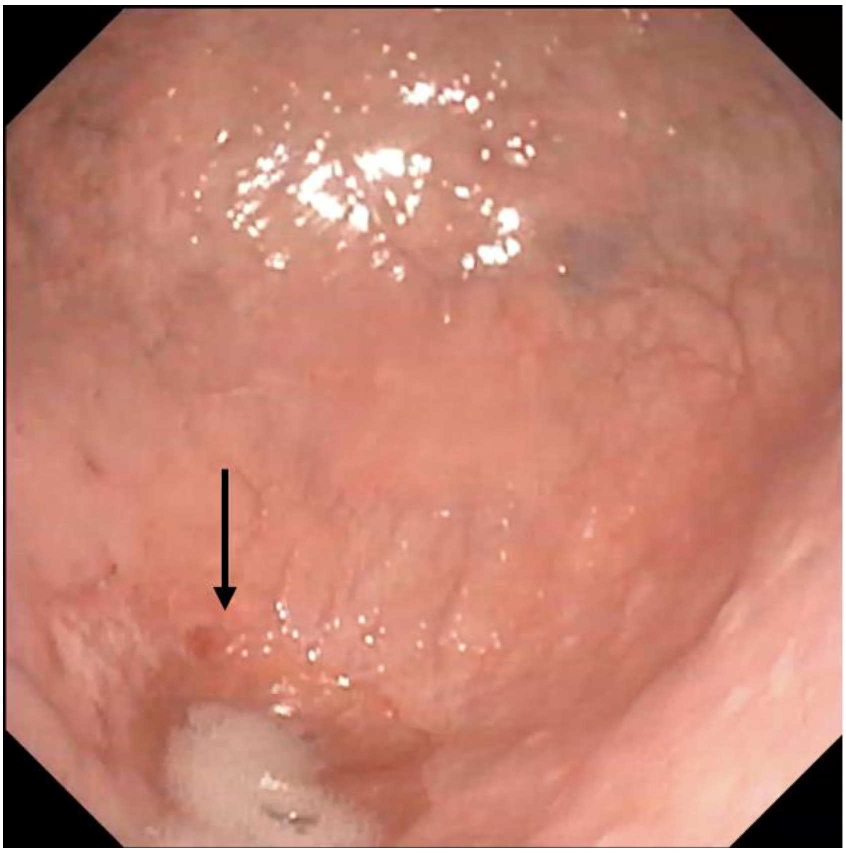

Supplement: Supplementary file 1 [file Data_Sheet_1.pdf]
